# Classification, angioarchitecture and treatment outcomes of medullary bridging vein-draining dural arteriovenous fistulas in the foramen magnum region: a multicenter study

**DOI:** 10.1007/s00234-024-03478-w

**Published:** 2024-10-12

**Authors:** Tomohiko Ozaki, Masafumi Hiramatsu, Hajime Nakamura, Yasunari Niimi, Shuichi Tanoue, Katsuhiro Mizutani, Ichiro Nakahara, Yuji Matsumaru, Yasushi Matsumoto, Timo Krings, Toshiyuki Fujinaka

**Affiliations:** 1 Department of Neurosurgery, National Hospital Organization, Osaka National Hospital, 2-1-14 Hoenzaka, Chuo-ku, Osaka, 540-0006 Japan; 2 Department of Neurosurgery, Osaka University Graduate School of Medicine, Suita, Japan; 3 Department of Neurological Surgery, Okayama University Graduate School of Medicine, Dentistry and Pharmaceutical Sciences, Okayama, Japan; 4 Department of Neuroendovascular Therapy, St. Luke’s International Hospital, Chuo-ku, Tokyo, Japan; 5 Department of Radiology, Kurume University School of Medicine, Kurume, Japan; 6 Department of Neurosurgery, Keio University School of Medicine, Shinjuku-ku, Tokyo, Japan; 7 Department of Comprehensive Strokology, Fujita Health University School of Medicine, Toyoake, Japan; 8 Division of Stroke Prevention and Treatment, Department of Neurosurgery, Faculty of Medicine, University of Tsukuba, Tsukuba, Japan; 9 Division of Development and Discovery of Interventional Therapy, Tohoku University, Sendai, Japan; 10 Division of Neuroradiology, Joint Department of Medical Imaging, Toronto Western Hospital, University Health Network, Toronto, ON Canada

**Keywords:** Dural arteriovenous fistula, Foramen Magnum, Transarterial embolization, Surgical obliteration, Bridging vein

## Abstract

**Purpose:**

This study aimed to classify medullary bridging vein-draining dural arteriovenous fistulas (MBV-DAVFs) located around the foramen magnum (FM) according to their location and characterize their angioarchitecture and treatment outcomes.

**Methods:**

Patients with MBV-DAVFs diagnosed between January 2013 and October 2022 were included. MBV-DAVFs were classified into four groups. Jugular vein-bridging vein (JV-BV) DAVF: located in proximity to jugular fossa, Anterior condylar vein (ACV)-BV DAVF: proximity to anterior condylar canal, Marginal sinus (MS)-BV DAVF: lateral surface of FM and Suboccipital cavernous sinus (SCS)-BV DAVF: proximity to dural penetration of vertebral artery.

**Results:**

Twenty patients were included, three JV-BV, four ACV-BV, three MS-BV and ten SCS-BV DAVFs, respectively. All groups showed male predominance. There were significant differences in main feeders between JV (jugular branch of ascending pharyngeal artery) and SCS group (C1 dural branch). Pial feeders from anterior spinal artery (ASA) or lateral spinal artery (LSA) were visualized in four SCS and one MS group. Drainage pattern did not differ between groups. Transarterial embolization (TAE) was performed in three, two, one and two cases and complete obliteration was obtained in 100%, 50%, 100% and 0% in JV, ACS, MS and SCS group, respectively. Successful interventions without major complications were finally obtained in 100%, 75%, 100%, and 40% in JV, ACS, MS and SCS group, respectively.

**Conclusion:**

JV-BV DAVFs were successfully treated using TAE alone. SCS-BV DAVFs were mainly fed by small C1 dural branches of vertebral artery often with pial feeders from ASA or LSA, and difficultly treated by TAE alone.

## Introduction

**Fig. 1:**
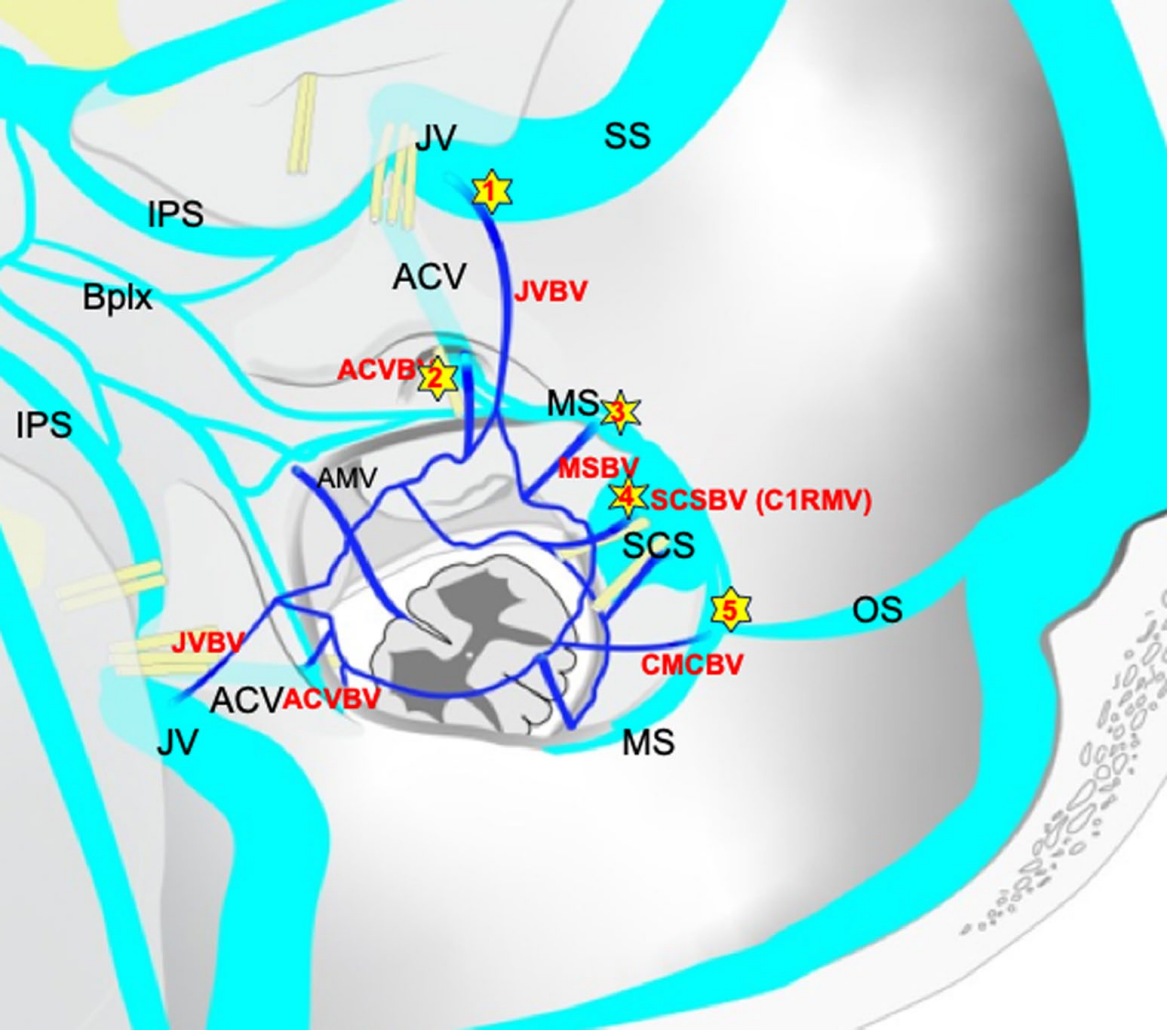
Schematic illustration of bridging vein (BV) and surrounding vessels around the foramen magnum: 1 shows jugular vein BV; 2, anterior condylar vein BV; 3, marginal sinus BV; 4, suboccipital cavernous sinus BV; 5, cerebellomedullary cistern BV. IPS = inferior petrosal sinus, Bplx = basilar plexus, JV = jugular vein, BV = bridging vein, ACV = anterior condylar vein, AMV = anterior medullary vein, MS = marginal sinus, SCS = suboccipital cavernous sinus, RMV = radiculomedullary vein, SS = sigmoid sinus, OS = occipital sinus

Dural arteriovenous fistulas of the foramen magnum region (FM-DAVFs) have been variably called DAVFs of the marginal sinus, craniocervical junction (CCJ), and anterior condylar confluence [1–7]. Because of their rarity and the complicated anatomy of the vasculature in this region, these FM-DAVFs are frequently misdiagnosed or mischaracterized. Mitsuhashi et al. reported a type of DAVF of the FM region which drains directly into a bridging vein (BV) of the medulla oblongata [8]. These medullary bridging vein-draining DAVFs (MBV-DAVFs) are more prevalent in older men and are clinically aggressive because of leptomeningeal venous reflux. They emphasized the importance of distinguishing these DAVFs from epidural and osteo-epidural ones because of differences in venous drainage, clinical characteristics, and treatment-related risks. MBVs drain perimedullary venous blood into the surrounding epidural plexus or the dural sinuses [8–10]. Because most course along the roots of the lower cranial nerves or C1, they were also called the “satellite veins of the cranial nerves” [9]. More recently, detailed anatomy of the MBVs around the FM has been investigated by 3D rotational angiography and cone-beam CT, and the MBVs were classified them into five subtypes according to the terminal location [11]. Those subtypes include anterior condylar vein(ACV)-BV, jugular foramen (JF)-BV, marginal sinus (MS)-BV, suboccipital cavernous sinus (SCS)-BV, and cerebellomedullary cistern (CMC)-BV (Fig. 1).

MBV-DAVFs may have characteristic features based on their specific location. In this retrospective multicenter cohort study, we classified medullary BV-draining DAVFs into the five groups according to the location of fistulas and evaluated typical angiographic features and treatment outcomes of these groups.

## Methods

This study protocol was approved by the institutional ethics committee of the participating centers and was performed in accordance with the committees’ guidelines.

### Patients

We retrospectively investigated consecutive patients with a DAVF draining only into a medullary BV and located between the level of the jugular tubercle and upper surface of C1 diagnosed between January 2013 and October 2022. Thirteen centers in Japan and one in Canada participated in the study. Patients with DAVFs draining into a dural sinus or emissary vein and those in whom three-dimensional angiographic imaging was insufficient to evaluate angioarchitecture in detail were excluded. Data regarding patient and clinical characteristics, imaging findings, treatment, treatment-related complications, and functional outcomes were recorded. Outcome was evaluated using the modified Rankin Scale (mRS) score.

### Evaluation of angioarchitecture

DAVF angioarchitecture including feeding arteries, draining veins, fistulous points were evaluated using biplanar digital subtraction angiography and three-dimensional rotational angiography with multiplanar reformation. Computed tomography and magnetic resonance imaging were also evaluated as a reference. All images were reviewed by a committee of eight readers (TO, HN, MH, HK, YN, ST, KM, TF) certified by the Japanese Society for Neuroendovascular Therapy to form a consensus interpretation. Type and origin of feeding arteries and presence of pial feeders and feeder aneurysms, draining pattern and presence of varices were evaluated. Draining pattern was classified according to direction of flow as cranial, caudal, or both. According to the BV classification previously reported [11], MBV-DAVFs were classified into five groups. (A) Jugular vein (JV)-BV DAVF: AVF located in proximity to the jugular fossa (on the superior and/or lateral surface of the jugular tuberculum), (B) ACV-BV DAVF: AVF located in proximity to the anterior condylar canal (on the inferior and/or medial surface of the jugular tuberculum), C): MS-BV DAVF: AVF located on the lateral surface of the foramen magnum, D) SCS-BV DAVF: AVF located at proximity to the dural penetration of the vertebral artery. E) CMC-BV DAVF: AVF located on the mid-dorsal surface of the FM (Fig. 1). Clinical symptoms, angioarchitectures, treatments, and outcomes were evaluated and compared in each group.

### Statistical analysis

Continuous variables were compared using the two-sided Student’s t-test or one-way analysis of variance with post-hoc Tukey–Kramer testing. Categorical variables were compared using the χ^2^ or Fisher’s exact tests. *P* < 0.05 was considered significant. Statistical analyses were conducted using JMP Pro 17 software (SAS Institute Inc, Cary, NC, USA).

## Results

Twenty patients were included for analysis (16 men and four women). Median age was 63.8 years (interquartile range [IQR], 54–73). Presentation was with congestive myelopathy in nine patients, subarachnoid hemorrhage in four, and cerebellar hemorrhage in one; six patients were asymptomatic. Details of patient characteristics were described in Table 1.

**Table 1:** Clinical characteristics and angioarchitecture

Case	Age	Sex	Clinical presentation	Arterial feeder	Drainage vein	Drainage direction	Varix
JV group							
1	68	M	No symptom	APA hgb, VA pma, OA jb	LMV, ASV	caudal & cranial	+
2	62	M	Myelopathy	APA jb, OA smb	ASV, PSV	caudal	-
3	65	M	Cerebellar hemorrhage	APA hgb & jb	LMV	cranial	-
ACV group							
4	63	M	SAH	APA hgb & jb	PLSV	lateral	+
5	46	M	Myelopathy	APA hgb	ASV, PSV	caudal	-
6	84	M	Myelopathy	VA C1db	ASV, PSV	caudal	-
7	49	M	Myelopathy	APA hgb & jb, VA C1db, PAA smb, MMA pb, PICA db	ASV, PSV, AMV, LMV	caudal & cranial	+
MS group							
8	54	M	Myelopathy	VA C1db	ASV	caudal	-
9	41	M	SAH	OA jb, LSA	AMV, ASV, PSV	caudal & cranial	-
10	75	M	SAH	APA jb, VA C1db	LMV	cranial	-
SCS group							
11	75	M	Myelopathy	APA hgb & jb, VA C1db, ASA	ASV, PSV	caudal	-
12	64	M	Myelopathy	VA C1db, and ama	ASV, PSV	caudal	-
13	54	F	No symptom	OA C1 db	AMV, LMV	cranial	-
14	74	F	Myelopathy	VA C1db and ama	ASV, AMV, LMV	caudal & cranial	-
15	68	M	SAH	VA C1db, ASA	ASV, AMV	caudal & cranial	-
16	73	M	No symptom	VA C1db, LSA	ASV, AMV	caudal & cranial	+
17	69	M	No symptom	VA C1db	ASV, AMV	caudal & cranial	-
18	47	F	No symptom	VA C1db, ASA	ASV, AMV, LMV	caudal & cranial	-
19	75	F	No symptom	VA C1db	ASV	caudal	-
20	70	M	Myelopathy	VA C1db, ASA	ASV	caudal	-

JV, Jugular vein; ACV, Anterior condylar vein; MS, Marginal sinus; SCS, Suboccipital cavernous sinus; APA, Ascending pharyngeal artery; VA, Vertebral artery; OA, Occipital artery; PAA, Posterior auricular artery; MMA, Middle meningeal artery; PICA, Posterior inferior cerebellar artery; LSA, Lateral spinal artery; ASA, Anterior spinal artery; hgb, Hypoglossal branch; pma, Posterior meningeal artery; jb, Jugular branch; smb, Stylomastoid branch; db, Dural branch; pb Petrosal branch; ama, Anterior meningeal artery; LMV; Lateral medullary vein; ASV, Anterior spinal vein; PSV, Posterior spinal vein; PLSV, Posterolateral spinal vein; AMV, Anterior medullary vein

### Classification (Fig. 1; Table 1)

Three, three, four and ten patients were classified into JV-BV DAVF (JV group), ACV-BV DAVF (ACV group), MS-BV DAVF (MS group) and SCS-BV DAVF (SCS group), respectively according to their fistulous point. There was no case classified into CMC-BV DAVF.

### Patient and clinical characteristics of each group (Table 2)

**Table 2:** Characteristics of DAVFs classified with the location of shunt point

No. Patients	JV group *n* = 3	ACV group *n* = 4	MS group *n* = 3	SCS group *n* = 10	*p* value
Age: mean ± SD	65.0 ± 17.3	60.5 ± 6.0	56.7 ± 6.9	66.9 ± 3.8	0.5747
Male: n (%)	3 (100)	4 (100)	3 (100)	6 (60)	0.3158
Aggressive symptoms: n (%)* Hemorrhagic presentation Myelopathy	2 (67) 1 (33) 1 (33)	4 (100) 1 (25) 3 (75)	3 (100) 2 (67) 1 (33)	5 (50) 1 (10) 4 (40)	0.2217 0.1970 0.6759
Right side of fistulous point: n (%)	0 (0)	2 (50)	2 (67)	6 (60)	0.4213
Feeders: no (%) VA C1dural branch ECA (including APA)* * APA* * OA* * Jugular branch from APA or OA Pial feeder from ASA or LSA					
	0 (0) 3 (100) 3 (100) 2 (67) 3 (100) 0 (0)	2 (50) 3 (75) 3 (75) 0 (0) 2 (50) 0 (0)	2 (67) 2 (67) 1 (50) 1 (50) 2 (66) 1 (33)	9 (90) 2 (22) 1 (11) 1 (11) 1 (11) 5 (50)	0.018 0.0347 0.0078 0.1059 0.031 0.050
Direction of venous drainage: n (%) Cranial Caudal Both	0.7962				
	1 (33) 1 (33) 1 (33)	0 (0) 2 (50) 1 (25)	1 (33) 1 (33) 1 (33)	1 (10) 4 (40) 5 (50)	
Draining veins: no (%) ASV PSV AMV LMV PLSV					
	2 (67) 1 (33) 0 (0) 2 (67) 0 (0)	3 (75) 3 (75) 1 (25) 1 (25) 1 (25)	2 (67) 1 (33) 1 (33) 1 (33) 0 (0)	9 (90) 2 (20) 6 (60) 3 (30) 0 (0)	
Venous varix: n (%)	1 (33)	2 (50)	0 (0)	1 (10)	0.2724

*Aggressive symptoms including intradural hemorrhage and myelopathy

**Lacking ECA injection in 1 case of MS group and SCS group in each

JV, Jugular vein; ACV, Anterior condylar vein; MS, Marginal sinus; SCS, Suboccipital cavernous sinus; VA, Vertebral artery; ECA, External carotid artery; APA, Ascending pharyngeal artery; OA, Occipital artery; ASA, Anterior spinal artery; LSA, Lateral spinal artery; ASV, Anterior spinal vein; PSV, Posterior spinal vein; AMV, Anterior medullary vein; LMV; Lateral medullary vein; PLSV, Posterolateral spinal vein

Age and gender did not significantly differ between four groups. Prevalence of myelopathy/intradural hemorrhage also did not significantly differ between four groups.

### Angioarchitecture (Table 2)

#### Feeding artery

MBV-DAVFs were fed by dural branches of the external carotid artery (ECA) alone (*n* = 5), the vertebral artery (VA) alone (*n* = 10), or both ECA and VA (*n* = 5). The most frequent feeders were jugular branch of the ascending pharyngeal artery (APA) or the occipital artery (OA) (*n* = 8), hypoglossal branch of the APA (*n* = 6), and the C1 dural branch of the VA (*n* = 13). Pial feeders via the vasa corona from the anterior spinal artery (ASA) (*n* = 3) or the lateral spinal artery (LSA) (*n* = 2) were seen in 5 patients.

In the JV group, all three cases were fed by dural branches of the ECA. In detail, all cases were fed by jugular branch of the APA or OA. The other feeders included hypoglossal branch (2/3), stylomastoid branch of the OA (1/3), and the posterior meningeal artery from the VA (1/3).

In the ACV group, three (75%) cases were fed by dural branches of the ECA and two (50%) were fed by the C1 dural branch of the VA. In detail, three were fed by hypoglossal branch of APA (3/4). The other feeders included jugular branch of the APA (2/4), petrosal branch of the middle meningeal artery (1/4), stylomastoid branch of the posterior auricular artery (1/4), and dural branch of the posterior inferior cerebellar artery (1/4).

In the MS group, two cases were fed by dural branches of the ECA (jugular branch of the APA or OA), and two cases were fed by the C1 dural branch of the VA. In detail, two cases were fed by jugular branch of the APA or OA. One case was fed by the lateral spinal artery (LSA).

In the SCS group, all 10 cases were fed by the C1 dural branch of the VA (9/10) or the OA (1/10). Only 1 case (10%) was fed by hypoglossal and jugular branch of the APA. Pial supply from the ASA (*n* = 4) or the LSA (*n* = 1) was seen in five patients (50%).

The frequency of ECA and APA (*p* = 0.0347 and 0.0078) significantly differed between groups. The jugular branch more frequently fed the JV group and less frequently fed the SCS group (*p* = 0.031), and the C1 dural branch of the VA more frequently fed the SCS group and less frequently fed the JV group with statistical significance (*p* = 0.008), The hypoglossal branch of the APA fed the ACV group more frequently than the other groups (*p* = 0.040). The frequency of pial feeder including ASA and LSA was significantly high in SCS group (50%) compared with other groups (0%, 0%, 33% of the JV group, ACV group and MS group, respectively, *p* = 0.050).

#### Drainage vein

The DAVFs drained through a medullary BV into the anterior or lateral medullary vein, and then cranially to the pontomesencephalic veins, lateral pontine vein, caudally to the anterior spinal vein (ASV) and/or the posterior spinal vein (PSV), or laterally via the transverse medullary vein and other medullary bridging veins. Direction of venous drainage were “caudal” in eight patients, “cranial” in three patients, both “caudal & cranial” in eight patients, and “lateral” in one patient. The draining pattern did not significantly differ between the groups (*p* = 0.7962). A varix was visualized along the drainage route in 4 patients.

### Risk factors associated with hemorrhagic presentation and myelopathy (Table 3)

**Table 3:** Risk factors associated with clinical presentation

Variable	Odds ratio	95% CI	*p* value
**Hemorrhagic presentation**			
Direction of venous drainage			
Cranial	9.33	0.62–123.6	0.0706
Cranial or Both	4.57	0.40–51.1	0.1945
Venous varix	1.00	0.08–12.56	1.0000
**Myelopathy**			
Direction of venous drainage			
Caudal	35	2.63–465.4	0.0018
Caudal or Both	NA	NA	0.0431

CI, Confidence interval; NA, Not applicable

Direction of the venous drainage is associated with the symptoms. Cases with caudal drainage more frequently presented myelopathy (7/8) and less frequently with hemorrhage (0/8). Myelopathy and hemorrhage were seen in two and two of 8 cases with “caudal&cranial” drainage and none and two of three cases with “cranial” drainage, respectively. Hemorrhagic presentation was not significantly associated with draining pattern, presence of varix, or location of fistula. Frequency of myelopathy was significantly higher in the cases with draining to caudal direction (including both direction) compared with cranial direction (56% vs. 0%, *p* = 0.0431).

### Treatment and outcomes (Table 4)

**Table 4:** Treatment and outcomes

Case	Treatment modality	AVF occlusion*	Complication**	Clinical outcome (mRS)
JV group				
1	TAE (OA jb) with NBCA	Complete	-	0
2	TAE (APA jb) with NBCA	Complete	-	4
3	TAE (APA hgb & jb) with NBCA	Complete	-	0
ACV group				
4	TAE (APA hgb) with NBCA	Complete	Transient XII nerve palsy	0
5	Surgery	Complete	-	1
6	Surgery	Complete	-	3
7	1. TAE (APA hgb &jb, VA C1 db) with NBCA 2. SRS	Residual	-	0
MS group				
8	Surgery	Complete	-	3
9	Surgery	Complete	-	0
10	TAE (APA hgb & jb, VA C1db) with NBCA	Complete	-	0
SCS group				
11	Surgery	Complete	-	4
12	Surgery	Complete	-	2
13	TAE (OA) with coil	Residual	-	0
14	1. TAE (VA C1db) with NBCA 2. Surgery	Residual (Complete: after surgery)	-	3
15	Conservative	NA	NA	6
16	Surgery	Complete	Stroke***	2
17	Surgery	Complete	Meningitis	1
18	Conservative	NA	NA	0
19	Conservative	NA	NA	0
20	Surgery	Complete	-	2

*Angiographical occlusion after initial treatment modality

**Complication after initial treatment modality

***Ischemic stroke due to intraoperative DSA

JV, Jugular vein; ACV, Anterior condylar vein; MS, Marginal sinus; SCS, Suboccipital cavernous sinus; TAE, Transarterial embolization; AVF, Arteriovenous fistula; mRS, Modified Rankin Scale; NBCA, n-butyl-2-cyanoacrylate; SRS, Stereotactic radiosurgery; NA, Not applicable; OA, Occipital artery; APA, Ascending pharyngeal artery; VA, Vertebral artery; jb, Jugular branch; hgb, Hypoglossal branch; db, Dural branch

In the JV group, all three cases were successfully treated by transarterial embolization (TAE) via the feeders from jugular branch and/or hypoglossal branch using N-butyl cyanoacrylate (NBCA) without any complications. There were no cases of recurrence.

In ACV group, two out of four cases were treated by TAE with NBCA via feeders from hypoglossal branch. One case showed complete occlusion of the DAVF, and the other showed residual DAVF after NBCA injection from multiple feeders. A minor complication of transient hypoglossal nerve palsy was observed after TAE in one case. The remaining two cases were successfully treated by open surgery.　 There were no cases of recurrence.

In MS group, all three cases were successfully treated by TAE using NBCA (*n* = 1) or open surgery (*n* = 2) without complications. There were no cases of recurrence.

In SCS group, only two cases (20%) were treated by TAE with coil (*n* = 1) or NBCA (*n* = 1). One showed incomplete occlusion of DAVF, and the other showed recurrence of DAVF within 10 months after TAE. Open surgery was applied in five cases. The five cases showed complete occlusion, but major complications of meningitis or cerebral infarction were observed in two of five cases. The remaining 3 cases were conservatively treated.

There were statistically significant differences in successful occlusion by transarterial embolization between the JV group and SCS group (*p* = 0.005). Successful intervention (complete occlusion of AVF without complications) by either TAE or open surgery was significantly less in SCS group (*p* = 0.030).

### Representative cases

#### DAVF with fistulous point located lateral to the jugular tubercle (JV-BV DAVF) with myelopathy treated by transarterial embolization (Fig. 2)

**Fig. 2:**
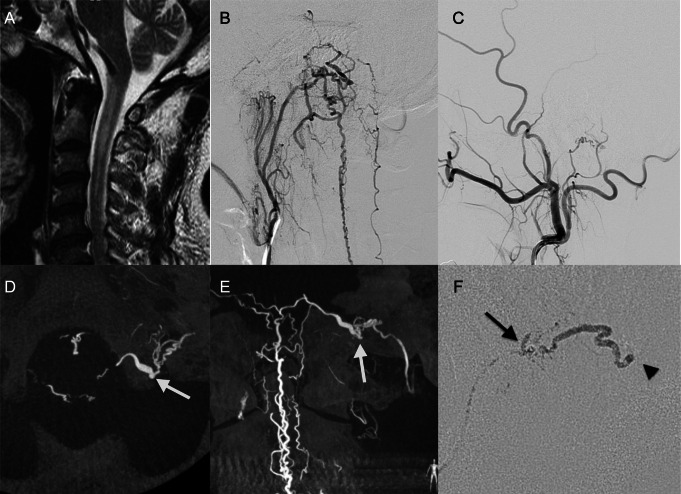
Representative case of DAVF with fistulous point located lateral to the jugular tubercle: JV-BV DAVF. (**A**) Sagittal T2 weighted image on admission showing high intensity area in the spinal cord at upper cervical levels. (**B**) Lateral view of the left ascending pharyngeal angiography showing jugular branch feeding a DAVF. The DAVF draining caudally into the anterior and posterior spinal veins. (**C**) Lateral view of the left external carotid angiography showing stylomastoid branch of the occipital artery feeding the DAVF. (**D**) Axial partial MIP image of 3D angiography showing fistulous point (arrow) located laterally to the jugular tubercle. (**E**) Coronal partial MIP image of 3D angiography showing fistulous point (arrow) close to jugular foramen. The dural feeders from the jugular branch of the APA converge on the fistulous point. The DAVF was classified as JV-BV DAVF. (**F**) Lateral view of DSA during NBCA injection from a microcatheter whose tip located in a dural feeder from the jugular branch of the APA. Arrow shows tip of the micro catheter. Arrowhead shows that NBCA cast reaches draining vein. The JV-BV DAVF was completely occluded with NBCA. JV = jugular vein, BV = bridging vein, DAVF = dural arteriovenous fistula, NBCA = n-butyl-2-cyanoacrylate

A 62-year-old man presented with bilateral leg weakness. Cervical magnetic resonance imaging showed central hyperintensity in the upper cervical spinal cord (Fig. 2A). Cerebral angiography showed a MBV- DAVF fed by the jugular branch of the ascending pharyngeal artery (APA) and the stylomastoid branch of the occipital artery (Fig. 2B, C). The fistulous point was located lateral to the jugular tubercle near the jugular fossa, and the DAVF was classified into JV group (Fig. 2D, E). The DAVF drained initially into a medullary BV, then went caudally to the anterior spinal vein (ASV) and perimedullary veins. A 1.3-Fr microcatheter was advanced into the feeder from the jugular branch of APA, and the DAVF was completely obliterated with 20% NBCA-lipiodol mixture without any complications (Fig. 2F). No recurrence has been observed for 120 months of follow-up periods.

#### DAVF with fistulous point located on the lateral surface of the foramen magnum (MS-BV DAVF) with subarachnoid hemorrhage treated by transarterial embolization (Fig. 3)

**Fig. 3:**
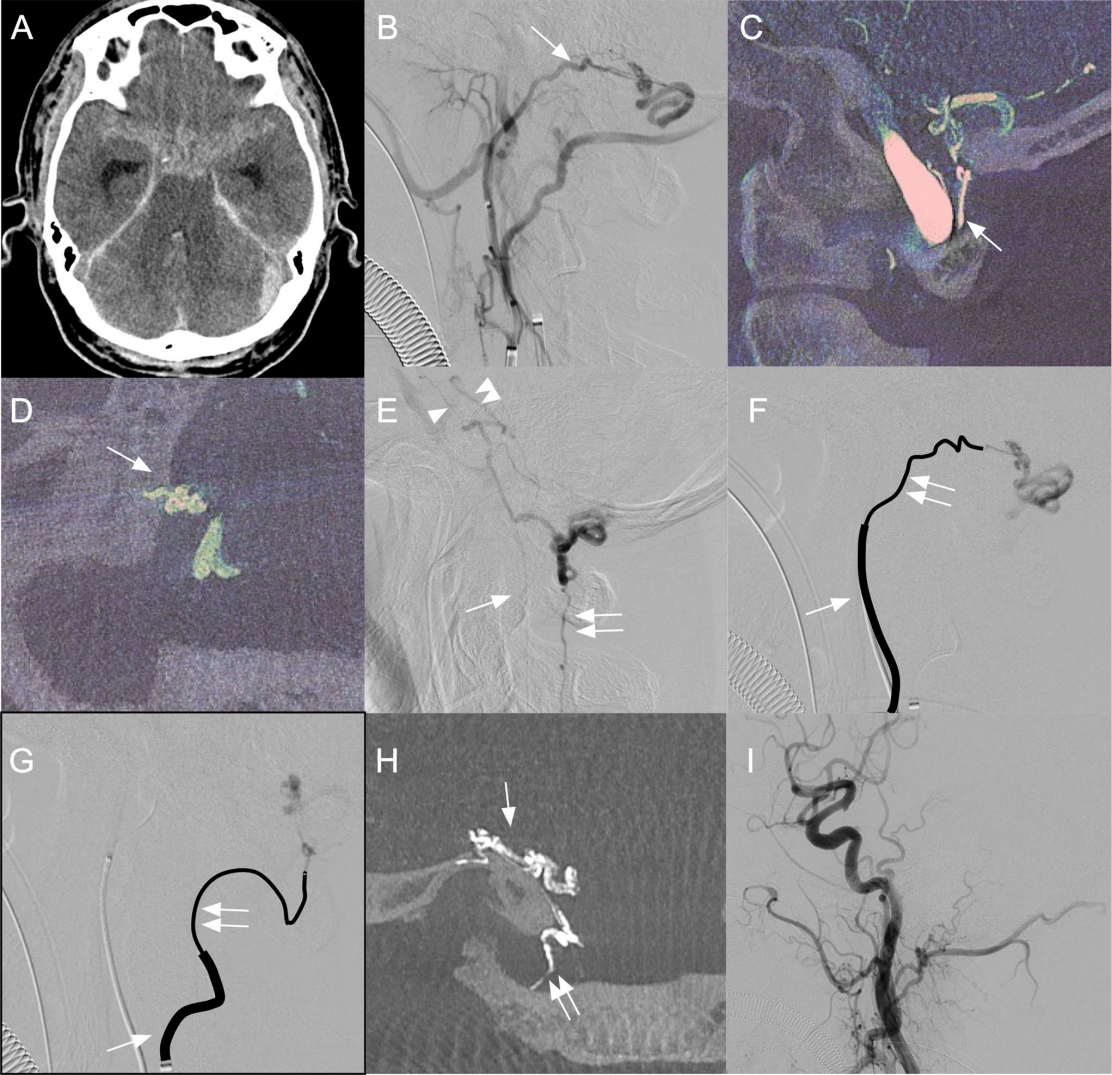
Representative case of the DAVF with fistulous point located on the lateral surface of the foramen magnum: MS-BV DAVF. (**A**) CT at admission showing subarachnoid hemorrhage. (**B**) Lateral view of the right external carotid angiography showing a DAVF at the level of foramen magnum. Arrow indicates jugular branch of the APA. (**C**) Sagittal MPR image of the right vertebral 3D angiography showing the C1 dural branch from VA (arrow) feeding the DAVF. (**D**) Axial MPR image of the right external carotid 3D angiography showing the feeder from the jugular branch continued to the fistulous points (arrow) on the lateral surface of the foramen magnum and then the medullary bridging vein. The DAVF was classified as MS-BV DAVF. (**E**) Late phase of the right external carotid angiography showing both cranial and caudal drainage routes from the MS-BV DAVF. Arrow, the anterior spinal vein; double arrows, the posterior spinal vein; arrowhead, the left lateral pontine vein; and double arrowheads, the right lateral pontine vein. (**F**) Lateral view of selective angiography with contrast injection from a microcatheter shows the tip located deeply in the feeder of the jugular branch of the right APA. Double arrows indicate a 1.3-Fr micro catheter which was coaxially introduced through a 2.7-Fr high-flow microcatheter (arrow). (**G**) Lateral view of selective angiography with contrast injection from another microcatheter located in the C1 dural branch of the right VA just before NBCA injection. Double arrows indicate a 1.3-Fr microcatheter which was advanced through a 3.4-Fr distal access catheter (arrow). (**H**) Coronal MPR image after NBCA injection from both feeders showing cast of the NBCA filling in the feeders and the drainage vein. Arrow shows the feeder from the jugular branch of the right APA and double arrow shows the C1 dural branch of the right VA. (**I**) Postoperative right common carotid angiography showing complete obliteration of the MS-BV DAVF. MS-BV DAVF = marginal sinus bridging vein dural arteriovenous fistula, MS = marginal sinus, BV = bridging vein, VA = vertebral artery, NBCA = n-butyl-2-cyanoacrylate

A 75-year-old woman was hospitalized because of subarachnoid hemorrhage (World Federation of Neurological Surgeons grade 2) (Fig. 3A). Cerebral angiography showed a MBV-DAVF fed by feeders from the jugular branch of the right APA and the C1 dural branch of the right VA (Fig. 3B, C). The fistulous point was located on the lateral surface of the foramen magnum (Fig. 3D), and the DAVF was classified as MS group. The DAVF was initially drained into a medullary BV and coronary veins then cranially via the lateral pontine vein into the right petrosal vein and caudally into the ASV and PSV (Fig. 3E). Two 4-Fr guiding sheaths were advanced into the right ECA and right VA with bilateral femoral arterial approach. Then, a 1.3-Fr (Carnelian Marvel S 1.3, Tokai Medical Products, Aichi, Japan)/2.7-Fr (Bishop HF, Piolax Medical Devices, Yokohama, Japan) coaxial microcatheter system was advanced into the jugular branch of the right APA as far as possible (Fig. 3F). Then, another 1.3-Fr microcatheter (DeFrictor, MEDICO’S Hirata, Osaka, Japan) was coaxially advanced through a 3.4-Fr distal access catheter (Technocrat Corporation, Aichi, Japan) to the dural branch of the right VA (Fig. 3G). NBCA was injected into the feeder from the jugular branch to partially fill the drainage vein beyond the fistulous point. Subsequently, NBCA was injected from the C1 dural branch of the right VA to completely obliterate the DAVF (Fig. 3H, I). No complications occurred. Recurrence has not been observed for 36 months of follow-up periods.

#### DAVF with fistulous point located at proximity to the dural penetration of the vertebral artery (SCS-BV DAVF) with myelopathy treated by open surgery (Fig. 4)

**Fig. 4:**
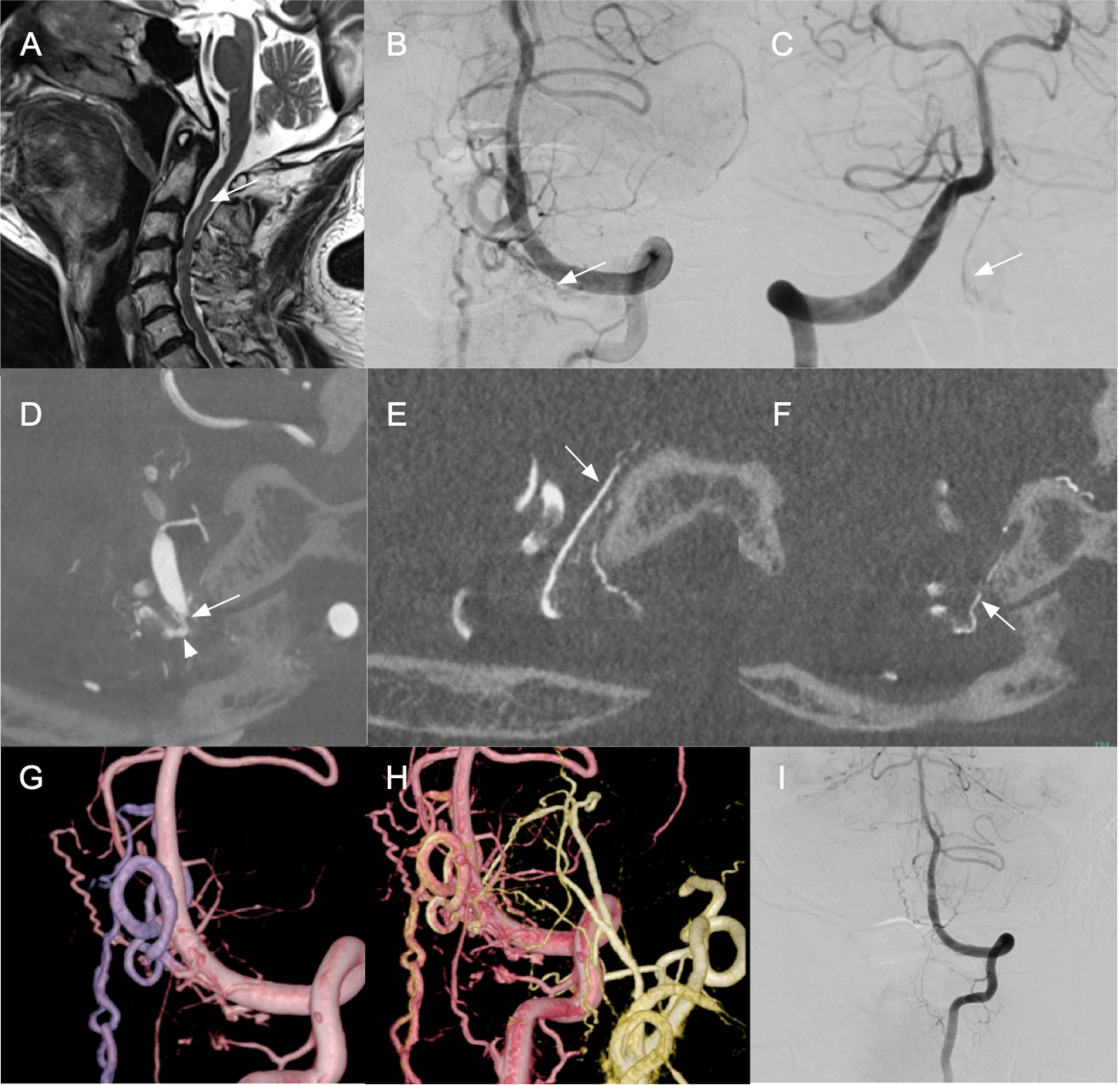
Representative case of DAVF with fistulous point located at proximity to the dural penetration of the vertebral artery: SCS-BV DAVF. (**A**) Sagittal T2 weighted image on admission showing high intensity area (arrow) in the spinal cord. (**B**) AP view of the left VAG showing C1 dural branch from the left VA (arrow) supplied to fistulous point. (**C**) AP view of the right vertebral angiography showing ASA (arrow) feed the SCS-BV DAVF. (**D**-**F**) Coronal MIP images of rotational angiography of the left VA (**D**), the left ECA (**E**, **F**) showing small C1 dural branches (arrow in **D**) of the VA, a feeder from the jugular branch (arrow in **E**) and a small feeder from the hypoglossal branch of the left APA converge on the fistulous point (arrowhead in **D**) at the level of O-C1. The DAVF was classified as the SCS-BV DAVF. (**G**)Volume rendering image of the left rotational vertebral angiography showing arteriovenous fistula supplied from dural branches of the left VA. (**H**) Fusion volume rendering image of the rotational angiographies of the left VA and the left ECA showing APA also supplied the DAVF at the same fistulous point with dural branch of the left VA. (**I**) Postoperative left vertebral angiography showing disappearance of the fistula. SCS = suboccipital cavernous sinus, BV = bridging vein, VA = vertebral artery, ECA = external carotid artery, APA = ascending pharyngeal artery, MIP = maximum intensity projection, ASA (anterior spinal artery)

A 75-year-old man presented with gait disorder and bladder disturbance. T2-weight MR image on admission showed high intensity area in the cervical spinal cord (Fig. 4A). The DAVF was mainly fed by the C1 dural branch from the left VA (Fig. 4B). It was also supplied by feeders from the left anterior spinal artery (Fig. 4C). Coronal MIP images of rotational angiography of the left VA (Fig. 4D) and the left ECA (Fig. 4E, F) showed small C1 dural branches of the VA (Fig. 4D), and a feeder from the jugular branch and a small feeder from the hypoglossal branch of the left APA (Fig. 4E, F) heading toward fistula at the level of O-C1. The DAVF was classified as the SCS-BV DAVF. The DAVF drained initially into a medullary BV, then cranially to the anterior medullary vein (AMV) and caudally into the PSV (Fig. 4G, H). Because of the higher risk of complications with endovascular embolization due to ASA feeding the DAVF, surgery was performed to interrupt the BV. Complete obliteration was achieved without complications (Fig. 4I). No recurrence has been observed for 4 months of follow-up periods.

## Discussion

In this retrospective multicenter cohort study, we classified medullary BV-draining DAVFs into the five groups according to the location of fistulas and evaluated typical angiographic features and treatment outcomes of these groups. The term of this bridging vein has been called such as bridging vein to the dural sinus [10], bridging vein around the foramen magnum [11] and medullary bridging vein [12]. These veins were identical with our medullary BV. These medullary BVs are running in the cistern　and connecting pial veins of peri-cervicomedullary junction, the longitudinal veins (anterior medullary vein and lateral medullary vein) and transverse veins of medulla and cervicomedullary junction, with the dural sinuses around foramen magnum.

MBV- DAVFs are rare, predominately occur in middle-aged men, and frequently present with hemorrhage or non-hemorrhagic focal neurological deficits [8, 12]. Mitsuhashi et al. reported a series of MBV- DAVFs with their clinical characteristics and treatment-related risks. More recently, Yoo et al. divided MBV- DAVFs according to their respective level of shunting into FM type and craniocervical junction (CCJ) type, and showed some differences in prevalence of feeding arteries and success rate of endovascular treatment between the two types of MBV-DAVFs [12]. According to their results, ECA supply was more frequent at the FM level (100%) than the CCJ (23.1%) level. Conversely, VA supply was more frequent at the CCJ level (100%) than the FM level (44.4%). Complete occlusion of DAVFs was less frequent in the CCJ type, with greater risk of ischemic complications.

This study proposed a novel classification of medullary bridging vein-draining DAVFs located around the FM according to the location of the AVF by precise evaluation of 3D angiographic images, and characterize their angioarchitecture and treatment outcomes. In our study, the location of the fistulous point was divided into five areas with reference to the location of BV around the FM [11]. Cadaver and imaging studies have shown that several types of BVs exist along with or apart from the lower cranial nerves and C1 nerve in 8–40% of individuals [9, 10, 13]. Those along the glossopharyngeal and vagal nerves drain into the jugular bulb [14] and those along the hypoglossal and C1 nerves drain into the ACV and SCS, respectively [9, 15]. The medullary BV may drain into the marginal sinus apart from the cranial nerves. The epidural venous plexus at the inferomedial portion of the jugular tubercle receives BVs draining into the marginal sinus or ACV. Similarly, the plexus at the lateral portion of the tubercle receives the BV to the jugular foramen and the one at the dural penetration zone of the VA receives the BV to the SCS. Therefore, the dural penetration zone of each type of medullary BV is thought to be the site of origin of each type of DAVF.

**Fig. 5:**
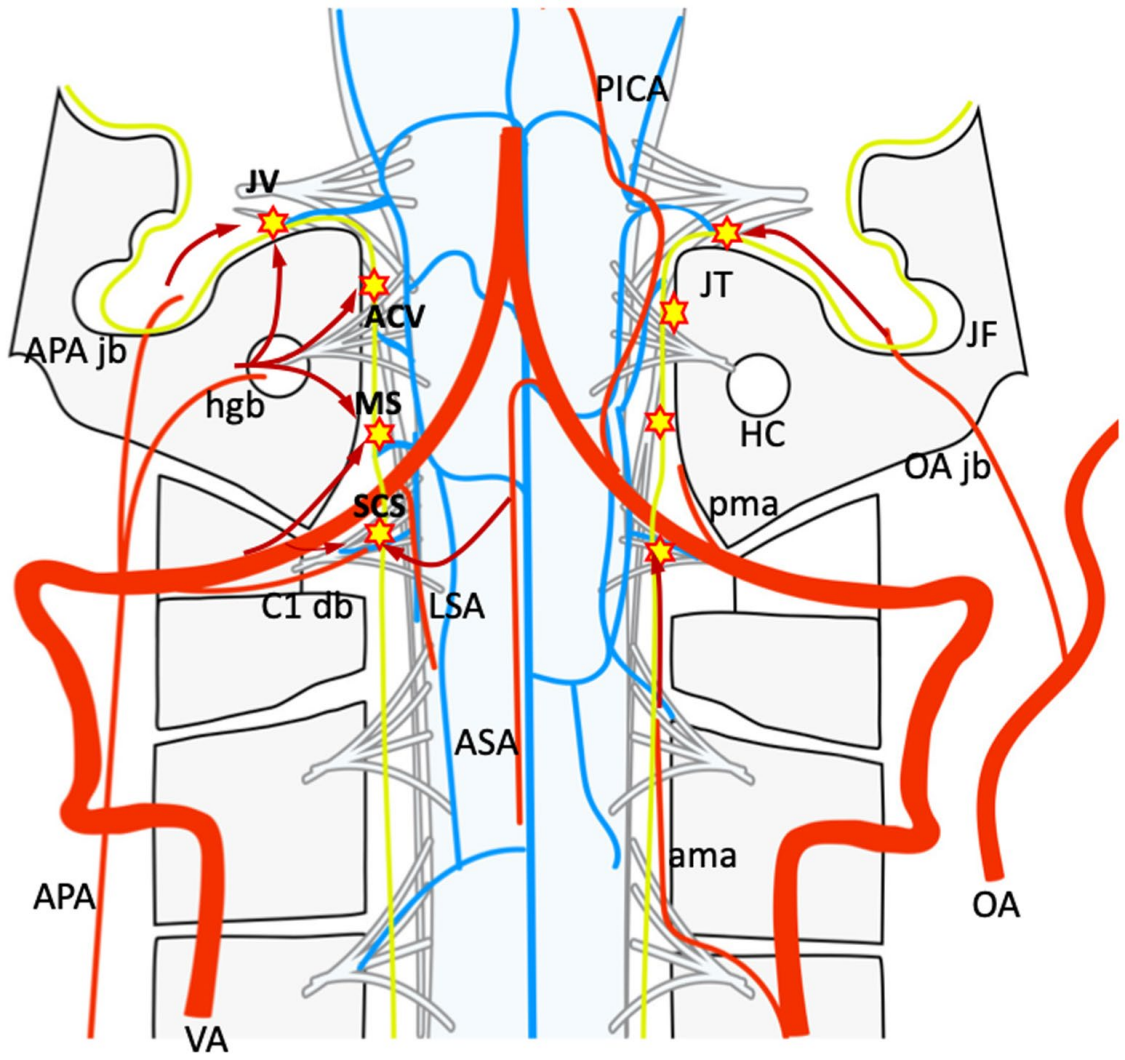
Schematic drawing of each group of MBV-DAVF and relevant feeders on coronal view. JB-BV DAVFs (JV) are usually fed by the jugular branch (jb)of the ascending pharyngeal artery (APA), ACV-BV DAVFs (ACV) are fed by the hypoglossal branch (hgb) of the APA and the C1 dural branch (db). MS-BV DAVFs (MS) are fed by both APA branches and the C1 dural branch. SCS-BV DAVFs (SCS) are fed by the C1 dural branch. The anterior meningeal artery (ama) and pial feeders from the anterior spinal artery (ASA) and lateral spinal artery (LSA) may also supply the SCS-BV DAVFs. PICA, posterior inferior cerebellar artery; pma, posterior meningeal artery; HGC, hypoglossal canal; JF, jugular fossa; OA, occipital artery

Frequent feeding arteries differed between groups probably due to anatomical distribution of the feeding arteries and location of the fistulous point (Fig. 5). JV-BV DAVFs were more frequently supplied by feeders from jugular branch of the APA, and ACV-BV DAVFs were supplied by hypoglossal branch of the APA. In contrast, SCS-BV DAVFs were mostly supplied by the C1 dural branches and the anterior meningeal artery from the VA. The MS-BV DAVFs were supplied by combined ECA and VA feeders. Regarding pial feeders from the ASA or LSA, they were more frequently observed in SCS group DAVFs (50%) than others. Yoo et al. also reported that MBV-DAVFs at the CCJ level had a possibility to have feeders from ASA (1 among 12 cases), on the other hand no FM level DAVFs had feeders from ASA in nine cases [12]. This is presumably due to the fistulous location of SCS group DAVFs. The fistulous point is located near dural penetration of the nerve root. The Radicular artery from ASA or LSA via vasa corona may reach the fistulous point. On the other hand, fistulous point of other group DAVFs may exist apart from the dural penetration of the nerve root. The short distance between the surface of the medulla oblongata and the dura matter at the CCJ level may also contribute the development of the pial feeders.

Venous drainage pattern did not differ between groups. It was associated with symptoms. Symptom of congestive myelopathy was associated with caudal drainage into the AVS and/or PSV probably due to developmental variation of the longitudinal spinal-brainstem drainage system such as anterior spinal and anterior medullary-anterior pontomesencephalic venous connection [16]. Poor development of those longitudinal channels and insufficient their epidural drainage may cause venous congestion in case of MBV-DAVFs. Although signal abnormality on medulla was detected in some cases, the symptoms of medulla, such as Wallenberg syndrome, inferior cranial nerve symptoms, did not appear in this study. Takai et al. reported that congestive myelopathy of CCJ DAVFs was characterized by ascending paralysis [17]. In their study including 27 CCJ DAVFs associated with myelopathy, patients with brainstem dysfunction were few (*n* = 5, 19%) compared to patients with tetraparesis (*n* = 18, 67%). They proposed a theory of this phenomenon. It presumably be associated with ischemia due to venous congestion of the anterior and lateral funiculus of the cervical cord by ventral descending venous drainage [17].

Open surgery is a curative treatment option for MBV-DAVF including CCJ DAVF, and some investigators showed good outcomes [18, 19], complications including hydrocephalus, meningitis, and spinal cord injury may occur [12]. According to recent advancements in endovascular technology, endovascular treatment has been spread rapidly in the world, and are often applied for MBV-DAVFs. Transvenous endovascular treatment is usually difficult to perform in MBV-DAVFs; therefore, TAE is usually selected for the first treatment option. However, TAE has a potential risk of a serious complication including spinal cord/medulla oblongata ischemia. Our study showed there are significant differences in efficacy of TAE between JV group and SCS group. The DAVFs could be completely occluded by TAE alone in all cases of JV group but none of SCS. This difference is probably due to differences of main feeder and coexistence of pial feeder in SCS group. JV-BV DAVF is usually fed by the jugular branch which usually runs non-tortuous course to the fistulous point. Therefore, it is often easier to advance a microcatheter close to the fistulous point. On the other hand, SCS-BV DAVF was always fed by the C1 dural branch which is usually small and runs tortuous course in a short distance to the fistulous point. Therefore, it is often difficult to advance a microcatheter to the safe point to inject liquid embolic materials. In addition, the C1 artery arising from the VA may supply the medulla via microscopic connections with the lateral spinal or posterior spinal artery and there is also potential risk to reflux into pial feeders once the shunting point is occluded [12, 16]. Open surgery may be better suited for the treatment of SCS-BV DAVFs. Open surgery was performed in six cases in our series. While the DAVF were completely obliterated in all six cases, major complication of meningitis and stroke occurred in 2 cases (33%). Thus, careful attention for the treatment strategy must be required for SCS-BV DAVF. Regarding ACV and MS groups, complete occlusion could be obtained by TAE alone in two of three cases (66%), and the other 4 cases were successfully treated by open surgery. The only one transit complication of transient hypoglossal nerve palsy was observed in one patient. The ACV-BV DAVF were frequently fed by the feeders from hypoglossal branch of the APA which usually runs more tortuous course through the hypoglossal canal, and super selective catheterization deeply via the hypoglossal branch is often difficult. Furthermore, the ACV- and MS- BV DAVFS were frequently fed by feeders from both the APA and the C1 dural branch of the VA, and therefore successful TAE may depend on vascular supply in each individual. Although the number of cases was small, TAE appears to be highly effective for the treatment of JV -BV DAVFs, if security margins to cranial nerve vascular supply is respected. Motebejane et al. reported in their series of FM DAVFs that all were managed using TAE with NBCA; in all cases, the catheter tip could be advanced beyond the hypoglossal or jugular foramen to prevent occlusion of the vasa nervosum [5]. Goss et al. reported no complications in their series of eight DAVFs treated using TAE via the APA [20]. Therefore, the APA appears to be a safe and effective route for TAE; however, the possibility of anastomoses with the internal carotid artery and VA must be kept in mind, as well as its supply to the lower cranial nerves [21, 22]. Thus, a liquid embolic agent that necessitates reflux or a plug (such as Onyx when injected without a Pressure cooker technique) may cause cranial nerve palsy.

This study has several limitations. It was retrospective in design. Therapeutic strategies, devices, and techniques differed among the participating centers. In addition, our sample size was small owing to the rarity of MBV-DAVFs. Further large-scale studies with a uniform treatment protocol are warranted to evaluate treatment outcomes.

## Conclusions

DAVF around the FM could be classified into four groups according to their location of fistulous point. JV-BV DAVFs were more frequently supplied by feeders from jugular branch of the APA and could be successfully treated by TAE. In contrast, SCS-BV DAVFs were supplied by the C1 dural branches of the VA, and often with the pial feeders. TAE of the SCS-BV DAVFs is more challenging. ACV-BV DAVF and MS-BV DAVF were fed by both APA branches and the C1 dural branch of the VA, and difficulty of TAE depends upon vascular supply in each individual. Venous drainage pattern did not differ between groups, but symptom of congestive myelopathy might be associated with caudal drainage pattern.

## Data Availability

The datasets generated and/or analyzed during the current study are not publicly available, but are available from the corresponding auther on reasonable request.
